# Quantitative analysis of intracellular communication and signaling errors in signaling networks

**DOI:** 10.1186/s12918-014-0089-z

**Published:** 2014-08-21

**Authors:** Iman Habibi, Effat S Emamian, Ali Abdi

**Affiliations:** 1 Center for Wireless Communications and Signal Processing Research, Department of Electrical and Computer Engineering and Department of Biological Sciences, New Jersey Institute of Technology, 323 King Blvd, Newark 07102, NJ, USA; 2 Advanced Technologies for Novel Therapeutics (ATNT), Enterprise Development Center, New Jersey Institute of Technology, 211 Warren St, Newark 07103, NJ, USA

**Keywords:** Cell signaling, Intracellular communication, Molecular networks, Signal transduction

## Abstract

**Background:**

Intracellular signaling networks transmit signals from the cell membrane to the nucleus, via biochemical interactions. The goal is to regulate some target molecules, to properly control the cell function. Regulation of the target molecules occurs through the communication of several intermediate molecules that convey specific signals originated from the cell membrane to the specific target outputs.

**Results:**

In this study we propose to model intracellular signaling network as communication channels. We define the fundamental concepts of transmission error and signaling capacity for intracellular signaling networks, and devise proper methods for computing these parameters. The developed systematic methodology quantitatively shows how the signals that ligands provide upon binding can be lost in a pathological signaling network, due to the presence of some dysfunctional molecules. We show the lost signals result in message transmission error, i.e., incorrect regulation of target proteins at the network output. Furthermore, we show how dysfunctional molecules affect the signaling capacity of signaling networks and how the contributions of signaling molecules to the signaling capacity and signaling errors can be computed. The proposed approach can quantify the role of dysfunctional signaling molecules in the development of the pathology. We present experimental data on caspese3 and T cell signaling networks to demonstrate the biological relevance of the developed method and its predictions.

**Conclusions:**

This study demonstrates how signal transmission and distortion in pathological signaling networks can be modeled and studied using the proposed methodology. The new methodology determines how much the functionality of molecules in a network can affect the signal transmission and regulation of the end molecules such as transcription factors. This can lead to the identification of novel critical molecules in signal transduction networks. Dysfunction of these critical molecules is likely to be associated with some complex human disorders. Such critical molecules have the potential to serve as proper targets for drug discovery.

## Background

Each cell in the human body includes many biomolecules, which continuously communicate with each other [1]. Intracellular signaling networks transmit signals from the cell membrane to the nucleus, via biochemical interactions. The goal is to regulate some target molecules, to properly control the cell function. This regulation of the target molecules occurs through communication of several intermediate molecules that convey specific signals originated from the cell membrane to the specific target outputs. Signaling networks have been studied in a number of different contexts [2]–[5]. From a communication system point of view, in this study we propose to model an intracellular signaling network as a communication channel. The message which is supposed to be communicated is a signal originated from the extracellular matrix that tells the cell what to do. The inputs of the communication channel could be ligands which upon binding to the cell surface, create a chain of interactions through some intermediate signaling molecules. This way the message is propagated towards the channel output, typically a target protein such as a transcription factor, to produce an appropriate response. Inputs and outputs of the channel can be considered as transmitters and receivers, respectively. In communication engineering, there are typically two types of channels, error-free and erroneous channels, which can correspond to functional and dysfunctional intracellular signaling networks, respectively. In an error-free communication channel, the message is transmitted without any error to the channel output. Signaling networks where all their molecules are functional can be considered as error-free channels, which allow the cell to correctly follow its input signals and function exactly the way it is supposed to. However, in an erroneous communication channel the message becomes distorted and signal might be lost. Delivery of the erroneous message to the channel output (typically a target protein) results in the malfunction of the cell. This may eventually result in a transition from the normal behavior (physiological condition) to a dysfunctional system (diseased or pathological condition).

Signal transmission in communication systems is accomplished via digital techniques, where different types of data and signals such as voice, music, image, video, text, etc., if not already in the digital format, are first digitized and converted to sequences of 0’s and 1’s [6],[7]. Digital communication in an error-free channel does not face any transmission error. For example, the digital input sequence 100010 can be transferred via an error-free cable channel to the destination, channel output, without any error (Additional file 1: Figure S1a). In an erroneous channel, however, there might be some transmission errors. For example, in a complex mobile wireless channel with significant amount of fading due to reflection and scattering via multiple paths, obstructed line-of-sight, etc., 0’s might be transmitted correctly, whereas 1’s might be incorrectly received as 0’s by a moving car (Additional file 1: Figure S1b).

In the area of systems biology, digital models and methods have been used for various purposes, and have also been verified using experimental data. The interested reader can refer to some review articles on this subject [8]–[13]. They have certain predictive and modeling capabilities that are particularly useful in large networks, where information on mechanistic details and kinetic parameters are not available. Some recent applications are discussed in [14]–[19].

Inspired by communication engineering and signal transmission concepts, in this paper we develop a systematic framework to quantitatively model how the signals that ligands provide upon binding can be lost in a pathological signaling network, due to the presence of some dysfunctional molecules. We also show how the lost signals result in message transmission error, i.e., incorrect regulation of target proteins at the channel output. Furthermore, we show how dysfunctional molecules affect the capacity of signaling networks and how the contribution of each signaling molecule to the signaling capacity and signaling errors can be computed. The proposed approach can quantify the role of dysfunctional signaling molecules in the development of the pathology.

## Results

### A simple pathological communication channel model for the Caspase3 network

**Figure 1:**
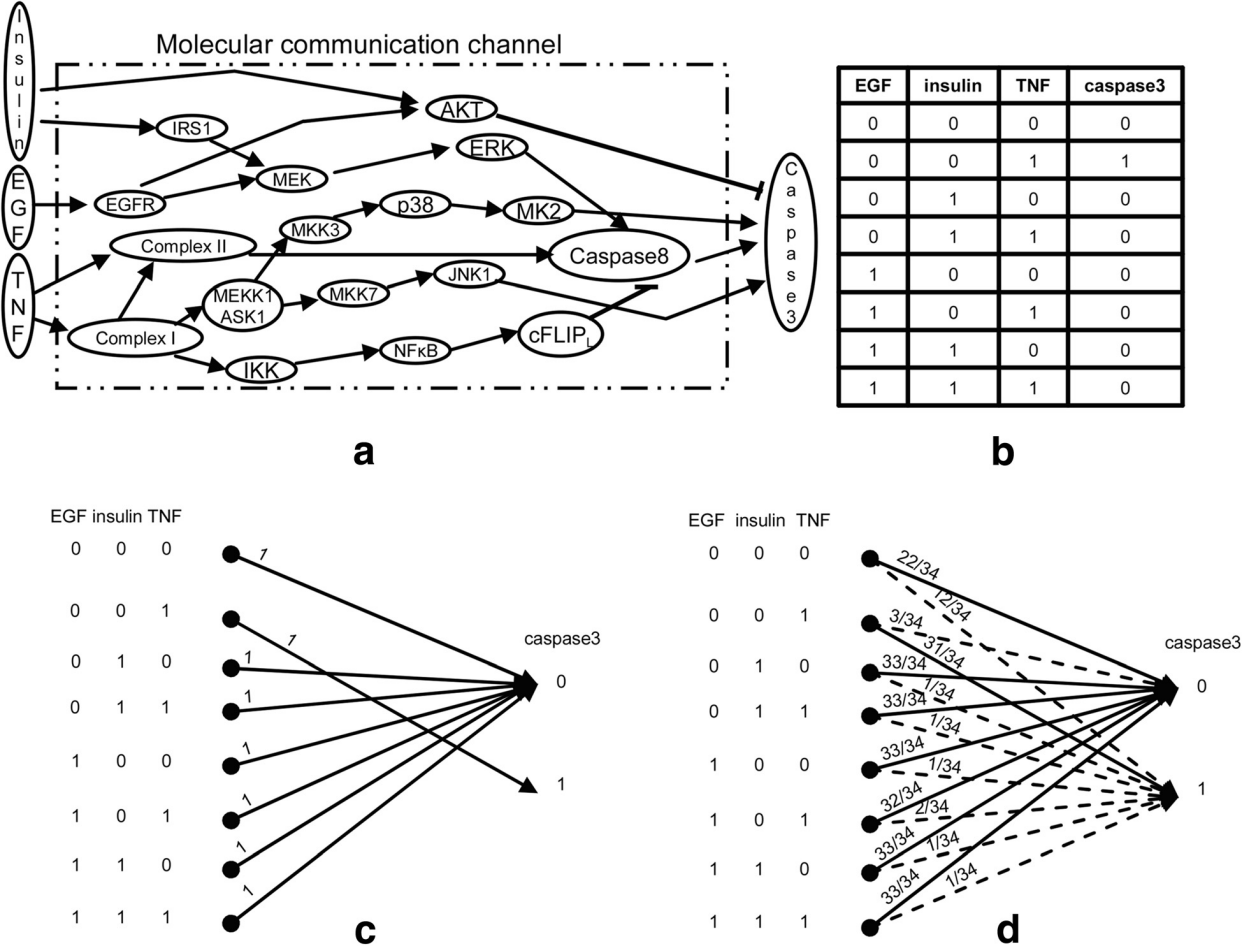
**The caspase3 network and its input-output characteristics and transition probability diagrams. (a)** The caspase3 communication channel. The channel input molecules are EGF, insulin and TNF, and the channel output molecule is caspase3. The molecule ComplexI within the channel includes TNFR and TRADD-RIP-TRAF2 [20], whereas the molecule ComplexII stands for TRADD-RIP-TRAF2 and FADD [20]. **(b)** The input–output relationships for the caspase3 normal channel. **(c)** Transition probability diagram for the normal caspase3 channel. Numbers above the arrows are transition probabilities. **(d)** Transition probability diagram for the pathological caspase3 channel where all the molecules in the channel are equally likely to be dysfunctional. Numbers above the arrows are transition probabilities.

Caspase3 is one of the most important molecules in the regulation of cell death (apoptosis) and cell survival. Caspase3 is a suitable molecule for the purpose of developing this approach for several reasons. This molecule has been extensively studied by several independent groups of scientists and the intracellular signaling molecules that regulate its activity are well characterized. Moreover, it is either in an active or inactive form. Caspases exist as inactive enzymes that undergo a proteolytic cleavage at conserved aspartic residues, to produce two subunits, large and small, that dimerize to form the active enzyme [1]. Dysfunction of the caspase network causes the failure of automated process of cell death and eventually results in a malignant transformation [1]. Signaling pathways from the input ligands EGF, insulin and TNF to the output caspase3 (Figure 1a) are extensively characterized and experimentally verified [20],[21]. Having the biological data and information from an independent group will validate the outcomes of this study, as discussed later. There are seventeen intermediate molecules between the inputs and the output, which constitute the communication channel of this network. The input–output relationships for the normal channel, i.e., when all the molecules in the channel are functional, are summarized in a table (Figure 1b), supported by the experimental findings of Janes *et al*. [20]. A value of 0 or 1 for a molecule means that it is either inactive or active, respectively [22]. Using the input–output relationships (Figure 1b), the channel transition probability diagram for the normal channel is obtained (Figure 1c).

Now we introduce a simple pathological communication channel model for the caspase3 network (Figure 1a). In this model, all the seventeen molecules in the channel are equally likely, i.e., with a probability of 1/17, to be dysfunctional. When a molecule is dysfunctional, its activity state does not change in response to its regulators. In this model we make a simple assumption that a dysfunctional molecule remains active, 1, or inactive, 0, with an equal probability of 1/2. This assumption could be easily changed to other possible probabilities of a molecule being dysfunctional, without affecting the proposed methodology. By calculating conditional probabilities (see Methods), the channel transition probability diagram for this pathological channel model can be constructed (Figure 1d). To understand the biological implications of the diagram, here we explain the arrows connecting 001 at the input to the 0 and 1 at the output, with the transition probabilities 3/34 and 31/34 written next to the arrows (other arrows can be similarly explained). Each transition probability is a conditional probability of the form *P*(caspase3|EGF, insulin, TNF) that we have calculated in (see Methods), using the total probability theorem [23]. Since there are 17 molecules in the channel and they are assumed to be equi-probable to be dysfunctional, the chance of each molecule to be dysfunctional becomes 1/17. A dysfunctional molecule is assumed to remain either always active or inactive, with a probability of 1/2. Therefore, the probability of each molecule in the channel to remain always active or inactive, irrespective of their input signals, is (1/17) × (1/2) = 1/34. According to our calculations (see Methods), in 3 out of 34 cases, caspase3 will be inactive, 0, when (EGF, insulin, TNF) = (0, 0, 1) in our pathological channel model. That is why this transition probability is 3/34. The probability of caspase3 to be active, when (EGF, insulin, TNF) = (0, 0, 1), is simply 1 − (3/34) = 31/34. The dashed arrow in the diagram (Figure 1d) means that when (EGF, insulin, TNF) = (0, 0, 1), the probability of capsase3 = 0 is 3/34, whereas the probability of capsase3 = 1 is 31/34. This implies that if, say, 100 molecules of TNF ligands bind to their receptors and activate them, then the number of inactive caspase3 molecules is 100 × 3/34 ≈ 9, whereas the number of activated caspase3 molecules is 100 × 31/34 ≈ 91. In this example, according to the channel transition probability diagram for the normal network (Figure 1c), in this example 100 caspase3 molecules should have been activated upon 100 TNF ligand bindings when EGF and insulin are inactive. However, in the pathological caspase3 channel, the remaining 9 inactive caspase3 molecules are the result of transmission errors occurred in the communication process because of the dysfunctional molecules in the signaling network.

Now we calculate the transmission error probability *P*_*e*_ for the caspase3 network. In the normal network (Figure 1c), input molecules correctly regulate the output molecule. This means that there is no transmission error probability, i.e., *P*_*e*,*normal channel*_ = 0. However, in the pathological network (Figure 1d), dysfunctional molecules do not allow the state of the output molecule to be correctly determined by the inputs. Using the total probability theorem and by considering all the error events, we have calculated the transmission error probability for the pathological caspase3 channel (Figure 1d), which is *P*_*e*,*abnormal channel*_ = 11/136 ≈ 0.08 (see Methods). This means out of one hundred (EGF, insulin, TNF) ligand bindings, on average eight caspase3 molecules will not be correctly regulated. From a signal transmission perspective, signal loss due to dysfunctional signaling molecules can be understood by noticing *C*_*normal channel*_ = 1, whereas *C*_*abnormal channel*_ = 0.69 (see Methods). We have calculated the signaling capacity by applying the algorithm in Methods to the transition probability channel matrix, obtained from the channel transition probability diagram of the caspase3 abnormal channel (Figure 1d). Note that the maximum signal content that the molecular network can convey from ligands to caspase3 is reduced because of the abnormalities in the network.

### A more general pathological communication channel model for the Caspase3 network

**Figure 2:**
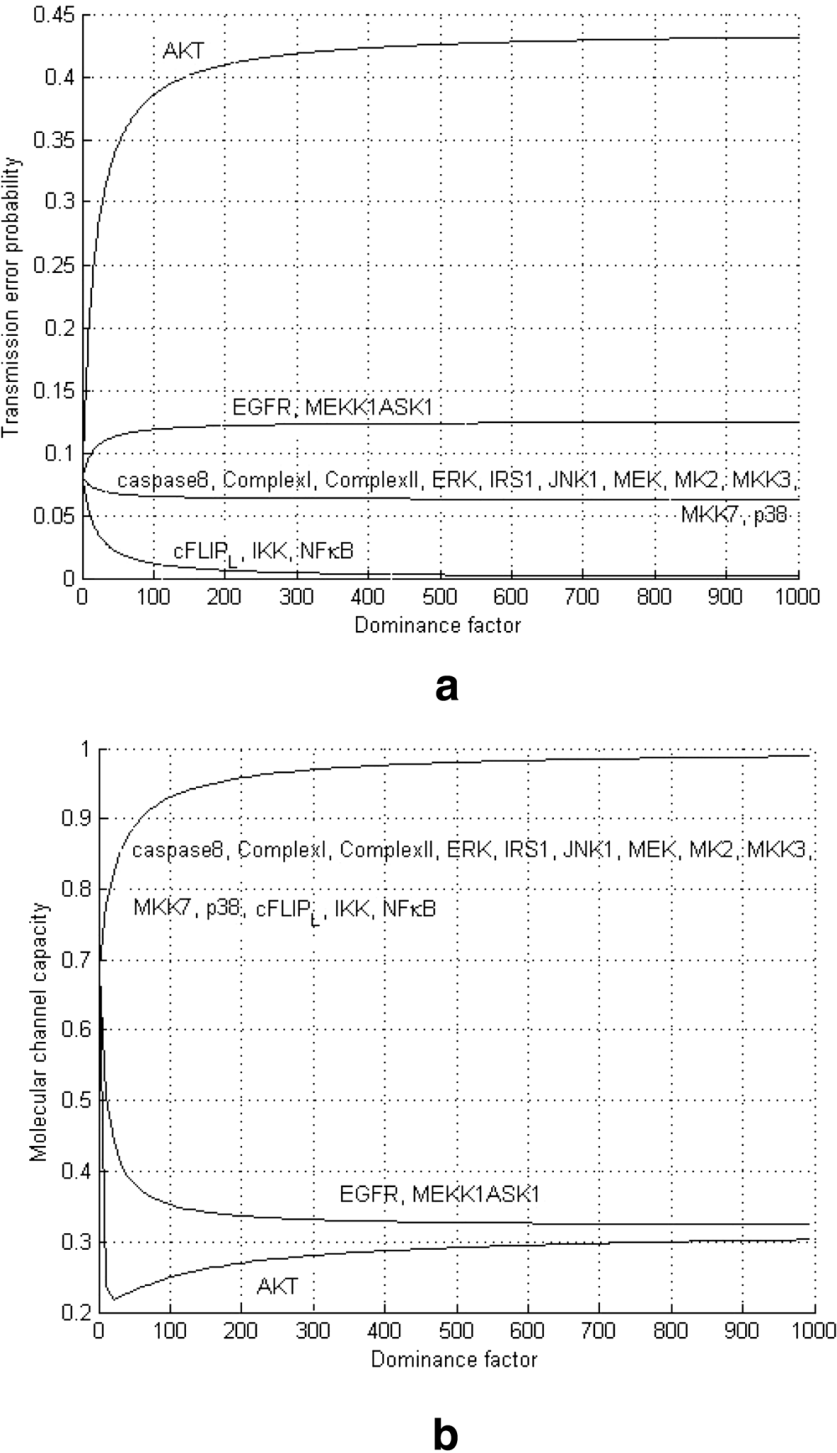
**The caspase3 network and its input-output characteristics and transition probability diagrams. (a)** Transmission error probability *P*_*e*_ versus the dominance factor *k* in the caspase3 communication channel. We have calculated transmission error probabilities using the total probability theorem and by considering all the error events (see Methods). The caspase3 network shows completely different behavior depending on the dominant dysfunctional molecule. For example, when AKT is the dominant molecule, *P*_*e*_ rapidly increases as the dominance factor *k* increases. This shows the critical role of AKT in signal transmission over this molecular communication channel. In contrast, other molecules, such as EGFR or MEKK1ASK1, cause a small increase in *P*_*e*_, which indicates that they have less impact on information transfer. The decrease of *P*_*e*_ for the rest of the molecules means that even if any of these molecules is dysfunctional with probability one, there will be no transmission error. **(b)** Caspase3 signaling capacity *C* versus the dominance factor *k*. Small values of *C* for AKT confirm the significant role of a dysfunctional AKT. Higher values of *C* for EGFR and MEKK1ASK1 mean that their dysfunction is less harmful to signal transmission than AKT. Large values of *C* for the rest of the molecules indicate their insignificance, when they are dysfunctional.

To explore how much each molecule contributes to channel transmission errors and signal loss, caused by dysfunctional molecules, now we introduce a more general channel model. In this model the probability of each molecule to be dysfunctional is *β*. However, there is one dominant molecule such that its dysfunctionality probability is *kβ*, *k* ≥ 1, where *k* is the dominance factor. In the model introduced earlier in the paper *k* = 1 but in this model the dominant molecule is more probable to be dysfunctional. Depending on which molecule in the channel (Figure 1a) is dominant, we obtain different channel transition probability diagrams, which result in different transmission error probabilities *P*_*e*_ (Figure 2a) and signaling capacities *C* (Figure 2b) (see Methods).

Similarly to the previous simpler channel model, we have calculated the transmission error probabilities (Figure 2a) using the total probability theorem and by considering all the error events (see Methods). Moreover, we have computed the signaling capacities (Figure 2b) by applying the algorithm in Methods to transition probability channel matrices. The results will be discussed in the Discussion Section.

### Experimental data to demonstrate signal transmission error

**Figure 3:**
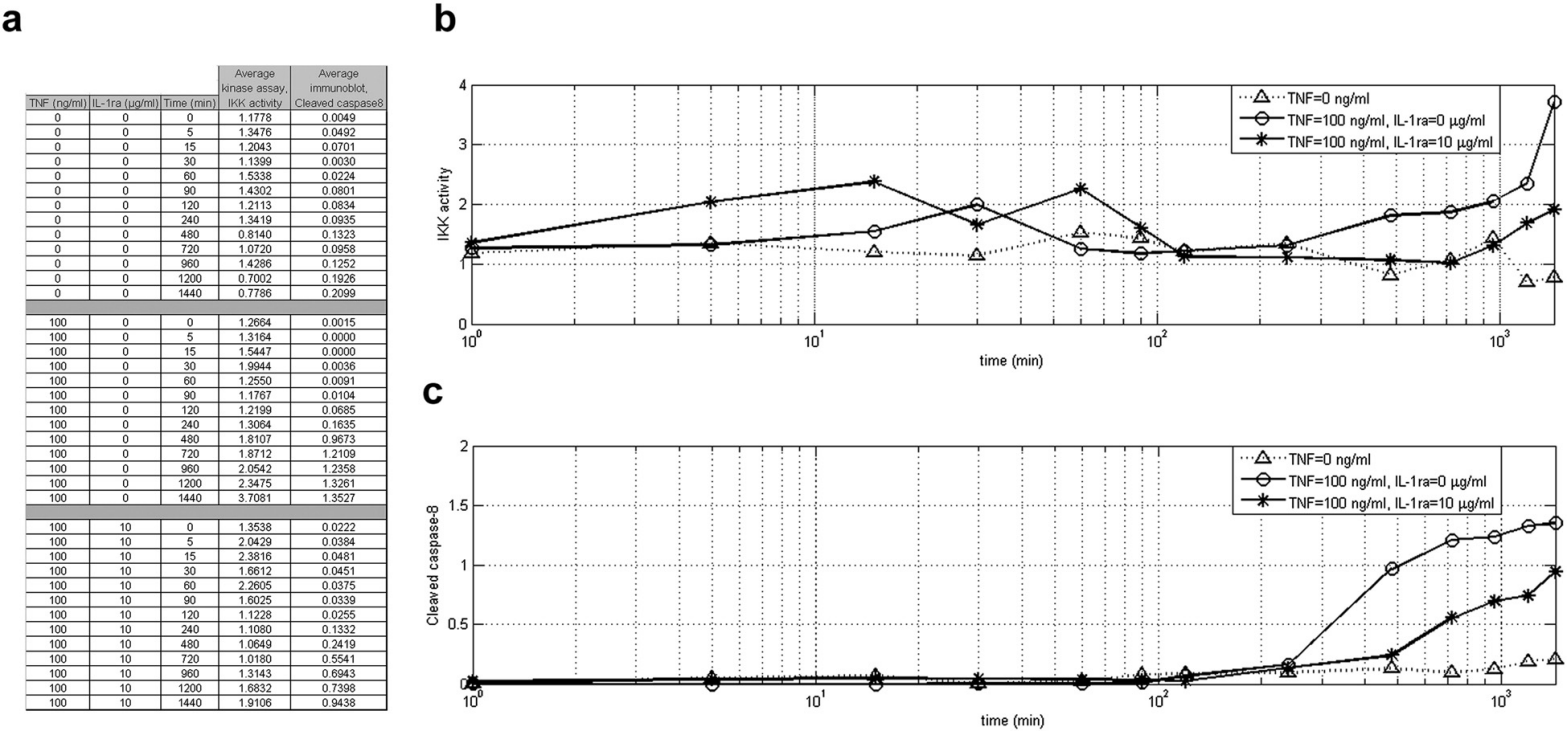
**Experimental IKK and caspase data. (a)** Measured biological data available in the Supplemental Data of Janes *et al*. [20]. For different concentrations of TNF (0 or 100 ng/ml) and IL-1ra (0 or 10 μg/ml), average IKK activity and cleaved caspase8 level are measured at thirteen time points, which start from 0 and end after 1440 minutes. **(b)** IKK activity versus time under three different conditions: no treatment (TNF = 0 ng/ml), treatment with TNF (TNF = 100 ng/ml, IL-1ra = 0 μg/ml), treatment with both TNF and IL-1ra (TNF = 100 ng/ml, IL-1ra = 10 μg/ml). From a communication system perspective, the “apoptosis” message is going to be transferred via IKK in the channel from the transmitter TNF to the receiver. Activation of TNF by increasing its concentration to 100 ng/ml can be viewed as TNF transmitting a signal. This signal is then propagated towards its downstream molecule IKK (Figure 1a). Activation of IKK eventually appears in long term, which means IKK has correctly received the signal from TNF. Adding IL-1ra, 10 μg/ml, acts as abnormality added to the communication channel, where IKK is located, and distorts the signal sent by TNF. This can be understood by looking at the decreased level of IKK activity in long term, which reflects the fact that IKK has not received the signal from TNF correctly. Hence, the “apoptosis” message has not been communicated successfully, and therefore the level of survival has increased [20]. **(c)** Cleaved caspase8 level versus time under three different conditions: no treatment (TNF = 0 ng/ml), treatment with TNF (TNF = 100 ng/ml, IL-1ra = 0 μg/ml), treatment with both TNF and IL-1ra (TNF = 100 ng/ml, IL-1ra = 10 μg/ml). See “Experimental data to demonstrate signal transmission error” in the Results section for further biological and communication engineering explanations.

We analyzed the experimental data of Janes *et al*. [20] (Figure 3a) to demonstrate the biological relevance of signal transmission error concept in a biological network. The data of Janes *et al*. [20] is a collection of protein levels or activity measurements of several molecules (Figure 3a) which are plotted versus time (Figure 3b and c). From a biological point of view and according to experiments data of Janes *et al.*[20], addition of TNF induces programmed cell death (apoptosis) through the activation of several mechanisms which are eventually reflected in the increased level of cleaved caspase8, a key caspase molecule that causes apoptosis (Figure 3c). However, by adding IL-1ra, the IL-1 receptor antagonist acting downstream to TNF, the apoptotic effect of TNF is significantly reduced [20]. This effect of IL-1ra is reflected in the decreased level of cleaved caspase8 (Figure 3c). The antagonistic effect of IL-1ra is reflected on the activity of the immediate downstream molecule IKK. As shown in Figure 3b, addition of IL-1ra caused an early fluctuation in the activity of IKK in the first 1.5 hours but caused a steady decrease in the activity of IKK, compared to the case without IL-1ra, after the first few hours. This continued to be the situation for several hours (Figure 3b). The decrease in IKK activity after long term treatment is nicely mirrored in the decreased level of cleaved caspase8 (Figure 3c) which again does not occur in the first 1.5 hours after treatment, but appears afterwards for several hours (compare IKK activity with cleaved caspase8 after 100 minutes of treatment with IL-1ra in Figure 3b and c).

From the proposed communication modeling point of view, the message is “apoptosis” that needs to be transmitted from the cell surface through a communication channel composed of several signaling molecules inside the cell. The transmitter is TNF, the receiver can be considered to be caspase8, and the channel is composed of the molecules in between (Figure 1a). Activation of TNF by increasing its concentration to 100 ng/ml can be viewed as TNF transmitting a signal. This signal is then propagated towards its downstream molecule IKK (Figure 1a). Activation of IKK eventually appears in long term (Figure 3b), which ultimately transmits the apoptosis signal, reflected in the increased level of cleaved calspase8 (Figure 3c). This can be interpreted as the correct reception of the apoptosis message sent by TNF in an error-free transmission. Adding IL-1ra, 10 μg/ml, acts as an abnormality added to the communication channel, which distorts the signal sent by TNF and causes transmission errors. This can be understood by looking at the decreased level of IKK activity in long term (Figure 3b). This eventually results in the decreased level of cleaved caspase8 (Figure 3c) which indicates incorrect reception of the apoptosis message. Because of this erroneous signal transmission, the level of survival has increased [20]. Therefore, using the data available in Janes *et al*. [20] one can see that signaling through normal and pathological molecular networks can be explained using signal transmission and reception concepts in communication channels.

### Communication engineering analysis of a large T cell signaling network

**Figure 4:**
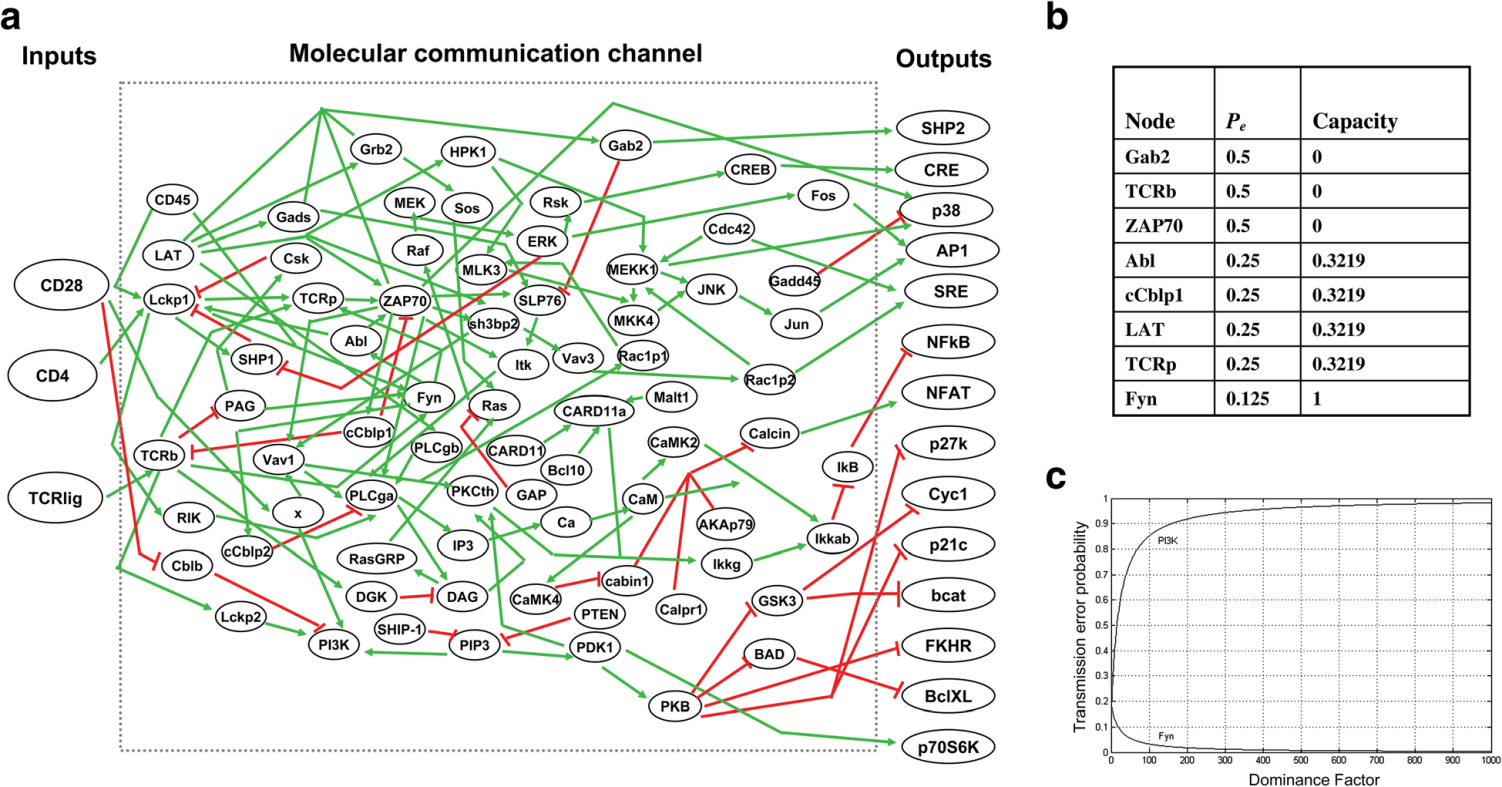
**Analysis of the T cell network. (a)** The T cell network [24]. The channel input molecules are TCR lig, CD4 and CD28, whereas the output molecules are AP1, bcat, BclXL, CRE, Cyc1, FKHR, NFκB, p21c, p27k, p38, p70S6K, SRE, NFAT and SHP2. Green arrows represent activatory interactions and red blunt lines show inhibitory interactions. This figure is intended to provide a general picture of the network. For specific details and regulatory mechanisms of each molecule, one can refer to the equations listed in Additional file 1: Table S1. **(b)** Values of transmission error probability *P*_*e*_ and capacity *C* are calculated for different molecules in the network with the output node SHP2, as an example output molecule. *P*_*e*_ and *C* values for those molecules not listed in this table are calculated as 0 and 1, respectively. See Additional file 1 for the list of these molecules. **(c)** Transmission error probability *P*_*e*_ versus the dominance factor *k* in the T cell communication channel for the two molecules PI3K and Fyn. When PI3K is the dominant dysfunctional molecule, *P*_*e*_ rapidly increases as the dominance factor *k* increases. This is in agreement with experimental data, which shows when PI3K is knocked out (inhibited with both Ly294002 and Wortmannin), PKB does not properly receive signals from the input molecules and remains inactive (its phosphorylation is blocked in human T cells) [24]. In contrast, the decrease of *P*_*e*_ when Fyn is the dominant dysfunctional molecule means that even if Fyn is dysfunctional with probability one, there will be no transmission error. This is consistent with the experimental observation that when Fyn is knocked out (Fyn-deficient and heterozygous splenic mouse T cells), stimulation of the input molecules still correctly regulates PKB [24].

Using the proposed bio-communication methodology developed in this study, we analyzed a large experimentally-verified model of a cellular network described by Saez-Rodriguez *et al.*[24]. This T cell network is composed of 94 different molecules, 123 interactions and multiple feedback loops, which give rise to a complex map of interactions based upon well-established findings from different studies on primary T cells (Figure 4a). The inputs of the T cell network [24] (Figure 4a) are TCR ligand (T cell receptor ligand) and two other receptors CD4 and CD28, whereas the outputs are AP1, bcat, BclXL, CRE, Cyc1, FKHR, NFκB, p21c, p27k, p38, p70S6K, SRE, NFAT and SHP2. This network is experimentally verified and characterized extensively [24]. There are seventy four intermediate molecules between the inputs and the outputs, which constitute the communication channel in the network (Figure 4a). There are four feedback loops in the network, regulating SHP1, cCblp1, PAG and Gab2. According to Saez-Rodriguez *et al.*[24] there are some molecules which regulate other molecules but their own regulation mechanisms are not clear: CARD11, GADD45, GAP, CD45, PTEN, BCL10, CDC42, MALT1, SHIP1, AKAP79 and CALPR1. We have similarly [24] included them in the network, with their states [24] specified in Additional file 1: Table S1. Here we present the results of the analysis of this network to show how the findings of proposed communication analysis method for the T cell network are biologically relevant and are also supported by the experimental findings of Saez-Rodriguez *et al.*[24] and other studies [21]–[24].

We studied the role and significance of each molecule in the T cell network (Figure 4a), in terms of their impacts on an output node, such as SHP2. Following the new methodology discussed above, we analyzed transmission error probabilities and capacities relevant to all molecules in this network, to identify molecules that have critical regulatory effect on the activity of the output node SHP2 in Figure 4b (see Methods). Similar to what is presented here for SHP2, transmission error probabilities and capacities can be computed for all other output molecules as well, and we only present the data for SHP2 as an example. As shown in Figure 4b, there are three molecules that are critically important for the accurate transmission of signals to output molecule SHP2. These molecules are Gab2, TCRb and ZAP70 that represent transmission error probabilities of 0.5 and capacities of 0. This means that for the correct propagation of message provided by inputs to the output molecule SHP2, the functions of these three molecules are very critical. In other words, the signaling capacity falls to zero if any of these molecules are dysfunctional. These findings are all biologically relevant and consistent with the experimental finding of Saez-Rodriguez *et al.* and other published literate. In more specific terms, critical regulatory effect of Gab2 on SHP2 has been established by several studies [25] and deregulation of this interaction is known to be associated with chronic myeloid leukemia (CML) [26]. TCRb has been known as a critical regulator of SHP2 for the past few years [27]. When we initially found the critical regulatory role of ZAP70 on SHP2 through this novel methodology, we thought this is a prediction of this analysis that has not been known in the past and we were trying to confirm this finding by experiments. However, later we noticed that another group has recently published convincing evidence for direct regulation of SHP2 by ZAP70, by affecting both enzymatic activity and phosphorylation levels of this molecule [28]. This recent paper, which nicely supports the prediction of our communication analysis method using extensive experimentation is the first paper that presents a direct evidence of critical regulatory role of ZAP70 on SHP2.

In this large network, we also considered two case studies, two molecules, to show that the results of the proposed method regarding signal transmission in signaling networks are consistent with experimental data [24]. The model described in Saez-Rodriguez *et al.* predicted the signaling events after antibody-mediated perturbation of CD28 and after genetic knockout of Fyn, which were subsequently experimentally validated. As shown in Figure 4c, comparative analysis of this network in terms of the importance of PI3K versus Fyn in the regulation of PKB (see Methods) identified that the molecule PI3K plays a key role in signal transmission from the input molecules TCR, CD4 and CD28, to the molecule PKB. This is because the transmission error probability increases, as PI3K becomes a dominant dysfunctional molecule (Figure 4c). This prediction is in agreement with the experimental data of Saez-Rodriguez *et al.*[24], which shows when PI3K is targeted (inhibited with both Ly294002 and Wortmannin), PKB does not properly receive signals from the input molecules TCR, CD4 and CD28 and remains inactive (its phosphorylation is blocked in human T cells) [24]. In contrast to the dominant role of PI3K, the same proposed communication analysis method has determined that the molecule Fyn does not cause signal transmission errors to PKB, when it is the dominant dysfunctional molecule (transmission error probability decreases as the dominance factor increases) (Figure 4c). This prediction is consistent with the experimental observation that when Fyn is targeted (Fyn-deficient and heterozygous splenic mouse T cells), stimulation of the input nodes TCR, CD4 and CD28 still correctly regulates PKB [24]. This means correct delivery of the input signals via the network to PKB, although Fyn is dysfunctional. Overall, the proposed method has been able to correctly identify and quantify different roles of PI3K and Fyn in a signal transduction network in T cells. These results are biologically relevant and consistent with experimental data [24].

## Discussion

In this study, we proposed a method to analyze pathological signaling networks and showed that the presence of dysfunctional molecules in these networks can cause signal loss. This results in message transmission error, i.e., incorrect regulation of the target protein at the network output. We also showed how the proposed method can be used to compute the level of contribution of dysfunctional signaling molecules to the signaling capacity loss of the network. Some critical molecules known to be involved in several disorders are correctly identified by this methodology, as discussed below.

As shown in Figure 2a, the caspase3 network shows completely different behavior depending on the dominant dysfunctional molecule. For example, when AKT is the dominant molecule, *P*_*e*_ rapidly increases as the dominance factor *k* increases (Figure 2a). This can reflect the critical role of AKT in signal transmission over this molecular communication channel. In contrast, other molecules, such as EGFR or MEKK1ASK1, cause a small increase in *P*_*e*_, which indicates that they have less impact on signal transfer. The decrease of *P*_*e*_ for the rest of the molecules means that even if any of these molecules is dysfunctional with probability one, there will be no transmission error (Figure 2a). Small values of the signaling capacity *C* for AKT (Figure 2b) confirm the significant role of a dysfunctional AKT. Higher values of *C* for EGFR and MEKK1ASK1 (Figure 2b) mean that their dysfunction is less harmful to signaling from inputs to caspase3, compared to AKT. Large values of *C* for the rest of the molecules indicate their insignificance in this specific network, when they are dysfunctional.

Transmission error probability introduced from a communication system engineering perspective in this paper is conceptually related to, but more general than the molecular vulnerability level [22]. In Abdi *et al.*[22], molecular networks were modeled using electronic circuit components and a simpler fault model was used, where the dysfunctionality probability of a faulty molecule was always one. The pathological communication model introduced in this paper is more general in the sense that it allows a molecule to be partially dysfunctional, i.e., its dysfunctionality probability can be changed from zero to one, using the dominance factor *k* of the model. In biological terms, we have several pathological conditions in which a dysfunctional molecule is partially expressed. For example, in schizophrenia, we see almost 50% reduction in total protein levels of AKT [29] in the brain of schizophrenic individuals, while several other signaling molecules in this pathway show normal levels [29]. This means that the dominance factor *k* is nearly 2 (=1/0.5) for AKT. Having the dominance factor *k* allows to examine the impact of each molecule on the network more precisely. With regard to the signaling capacity introduced and calculated in this paper, we can say it shows the maximum signal content that a signaling network can convey to correctly regulate target molecules such as transcription factors. If the presence of some dysfunctional molecules in a network deteriorates the capacity significantly, then the network fails to regulate its output molecules. Therefore, one can use signaling capacity as an index to characterize the role of molecules in a network. A molecule which can considerably decrease the capacity if it is dysfunctional, can be the cause of significant signal loss in the network, and might be correlated with some diseases. The computational results show that a dysfunctional AKT provides the lowest signaling capacity (Figure 2b). This can be related to the crucial role of AKT in the pathologic process of a number of common human malignancies [30],[31], including breast cancer, prostate cancer, lung cancer, gastrointestinal tumors, pancreatic cancer, hepatocellular carcinoma, thyroid cancer, and CNS malignancies (such as glioblastoma and gliomas), and other disorders such as schizophrenia [29].

The proposed method can be extended to handle multiple simultaneous dysfunctional molecules. However, in practice, due to the large number of all possible multiple cases, it is not feasible to check all the possible cases. For example, in a network with 20 molecules, the total number of single dysfunctional molecules is 20. In the same network, however, the total number of all possible single and multiple dysfunctional molecules together is 2^20^ − 1 = 1, 048, 575 (!). Besides the high computational complexity involved, the results might be non-conclusive, due to the extremely large number of possible situations. To avoid this, one can consider a subset of multiple dysfunctional molecules. For example, one can start with pairs of simultaneous dysfunctional molecules, to obtain transition probability diagrams, calculate transmission error probabilities, etc., following the proposed method. Such study can determine which pairs of molecules are the most critical ones for signal loss in the network, when both molecules are dysfunctional. Due to space limitation, we are not including such studies in the paper in detail. However, here we provide few case studies in the caspase3 network, to demonstrate the feasibility of analyzing multiple dysfunctional molecules. For example, when {AKT,JNK1} is the dominant dysfunctional pair and the remaining molecules are functional, we obtain the transmission error probability to be *P*_*e*_({AKT, JNK1}) = 0.4688. Comparison of this result with *P*_*e*_’s obtained when only a single dominant molecule is dysfunctional reveals some notable characteristics. When {AKT} and {JNK1} are dominant individual dysfunctional molecules, equations (20) and (23) result in *P*_*e*_({AKT}) = 0.4375 and *P*_*e*_({JNK1}) = 0.0625, respectively, with *k* = ∞. However, if they are both dysfunctional simultaneously, *P*_*e*_ increases to 0.4688. This indicates that simultaneous dysfunction of two molecules could be more harmful to the entire network, compared to their individual malfunction. The same observation is made regarding the dysfunction of {EGFR}, {IRS1} and {EGFR, IRS1}, since *P*_*e*_({EGFR}) = 0.125, *P*_*e*_({IRS1}) = 0.0625 and *P*_*e*_({EGFR, IRS1}) = 0.1875.

## Conclusions

In summary, this study demonstrates how signaling networks can be modeled as communication systems. This approach takes advantage of the concepts of communication engineering and signal transmission, to model and analyze networks with dysfunctional molecules as pathological communication channels. The fundamental concepts of transmission error probability and signaling capacity are defined for intracellular signaling networks, and proper methods for computing these parameters are developed. Application of the proposed methods to the caspese3 and T cell signaling networks provided biologically relevant findings which are also consistent with experimental data. Overall, this study shows how signal transmission and distortion in pathological signaling networks can be modeled and studied using a new communication analysis framework. The proposed method allows to determine how much the functionality of each molecule in the network affects signal transmission and regulation of end molecules such as transcription factors. This can lead to the identification of novel critical molecules in signal transduction networks. Dysfunction of these critical molecules is likely to be associated with some complex human disorders. Such critical molecules have the potential to serve as proper targets for drug discovery [32].

## Methods

### Definitions and generic description of the methods

In general, a molecular network consists of several inputs, intermediate molecules and output molecules. To be able to analyze the network (also known as channel here), first we need to specify the equations that determine the channel outputs in terms of the channel inputs. Then we use them to calculate the transition probability channel matrix for the network. The size of the matrix depends on the number of network inputs and outputs. Each element of the transition probability channel matrix specifies *P*(outputs|inputs), the probability of the outputs to be 0 or 1, conditioned on the 0 or 1 states of the inputs. The transition probability channel matrix **M** for a general molecular network with *m* inputs *x*_1_, *x*_2_, …, *x*_*m*_ and *n* outputs *y*_1_, *y*_2_, …, *y*_*n*_ is given in (1):

(1) $$\begin{array}{l} \;\mathrm{Inputs}\;x_{1},\;x_{2},\;\ldots ,\;x_{m}\\ M=\left[\begin{array}{cccc} P\left(0\cdots 00|0\cdots 00\right) & P\left(0\cdots 01|0\cdots 00\right) & \cdots & P\left(1\cdots 11|0\cdots 00\right)\\ P\left(0\cdots 00|0\cdots 01\right) & P\left(0\cdots 01|0\cdots 01\right) & \cdots & P\left(1\cdots 11|0\cdots 01\right)\\ \vdots & \vdots & \ddots & \vdots \\ P\left(0\cdots 00|1\cdots 11\right) & P\left(0\cdots 01|1\cdots 11\right) & \cdots & P\left(1\cdots 11|1\cdots 11\right) \end{array}\right]\;\begin{array}{c} 0\cdots 00\\ 0\cdots 01\\ \vdots \\ 1\cdots 11 \end{array}\\ \mathrm{Outputs}\;y_{1},\;y_{2},\;\ldots ,\;y_{n}\;\begin{array}{cccc} 0\cdots 00\; & 0\cdots 01 & \;\cdots \; & \;1\cdots 11 \end{array} \end{array}$$

For any given set of 0 and 1 values for the network inputs, each element of the matrix in (1) gives the probability of the network outputs to take a certain sequence of 0 and 1 values. In a pathological network with some dysfunctional molecules, some network output states might be erroneous (different from normal network outputs). Therefore, some elements of the transition probability channel matrix might be different under normal and pathological conditions. By taking all these differences into account and using the total probability theorem [23], transmission error probability which quantifies network signaling errors can be determined. In other parts of the Methods section and also in Additional file 1, the above concepts and methods are applied to several networks.

Signaling capacity is another metric that we use to analysis signaling networks. To calculate the capacity of a molecular network, first we need to define entropy, equivocation and mutual information. Entropy is a measure of uncertainly in a random variable and shows its unpredictably. For a discrete random variable *X*, entropy is mathematically defined by Shannon as follows [33]:

(2) $$H\left(X\right)=-\sum_{x\in X}p\left(x\right)\log_{2}p\left(x\right),$$

where *p*(*x*) is the probability of the state *x* ∈ *X*. Equivocation or the conditional entropy *H*(*X*|*Y*) is the remaining uncertainty about *X,* after observing another random variable *Y.* Its definition is given by [33]:

(3) $$H\left(X|Y\right)=-\sum_{y\in Y}\left(p\left(y\right)\sum_{x\in X}p\left(x|y\right)\log_{2}p\left(x|y\right)\right),$$

where *p*(. |.) is the conditional probability. When the input of a system is random, we are uncertain about the system input. However, by observing the system output we can reduce this uncertainly and gain information. Based on this concept, mutual information between the input *X* and the output *Y* is defined as [33]:

(4) $$I\left(X;Y\right)=H\left(X\right)-H\left(X|Y\right)=-\sum_{x\in X}p\left(x\right)\log_{2}p\left(x\right)+\sum_{y\in Y}\left(p\left(y\right)\sum_{x\in X}p\left(x|y\right)\log_{2}p\left(x|y\right)\right).$$

The amount of information that is provided to the input of a molecular signaling system can be considered as the input entropy. Equivocation can be considered as a measure of the information loss in a signaling system. The difference between these two is the mutual information between system input and output. As can be observed in (4), mutual information depends on the input probability distribution. However, in many applications we typically prefer to have a metric which is related only to the system itself. This is why the system capacity was proposed by Shannon as an intrinsic property of the system. By definition, capacity is equal to the maximized mutual information over all possible input distributions [33]:

(5) $$C\left(X;Y\right)=\underset{p\left(x\right)}{\sup}I\left(X;Y\right),$$

where sup stands for supremum.

For cell signaling networks, typically it is not straightforward to determine the input distribution. However, we can determine the mutual information as a function of input distribution, using the input–output relations. Then we can find the particular input distribution which maximizes the mutual information. The maximum mutual information is the system capacity.

### Method for calculating channel transition probabilities in the normal caspase3 network

A channel transition probability for the channel of Figure 1a is a conditional probability of the form *P*(caspase3|EGF, insulin, TNF). It specifies the likelihood of the output to be 0 or 1, conditioned on the 0/1 states of the inputs. When the caspase3 network in Figure 1a operates normally, i.e., all the molecules are functional, transition probabilities can be easily determined using the input–output table of the network (Figure 1b). According to Figure 1b, when (EGF, insulin, TNF) = (0, 0, 1), i.e., EGF and insulin are inactive whereas TNF is active, capsase3 becomes active, i.e., capsase3 = 1. This means that *P*(caspase3 = 1|EGF = 0, insulin = 0, TNF = 1) = 1 and *P*(caspase3 = 0|EGF = 0, insulin = 0, TNF = 1) = 0. For the other seven input combinations, caspase3 is inactive, i.e., capsase3 = 0. This implies that for these seven cases we have *P*(caspase3 = 0|EGF, insulin, TNF) = 1 and *P*(caspase3 = 1|EGF, insulin, TNF) = 0. All these channel transition probabilities are graphically shown in Figure 1c, as well as the following transition probability channel matrix **M**:

(6) $$\begin{array}{l} \;\mathrm{EGF},\;\mathrm{insulin},\;\mathrm{TNF}\\ M_{\mathrm{normal}\;\mathrm{channel}}=\left[\begin{array}{cc} P\left(0|000\right) & P\left(1|000\right)\\ P\left(0|001\right) & P\left(1|001\right)\\ P\left(0|010\right) & P\left(1|010\right)\\ P\left(0|011\right) & P\left(1|011\right)\\ P\left(0|100\right) & P\left(1|100\right)\\ P\left(0|101\right) & P\left(1|101\right)\\ P\left(0|110\right) & P\left(1|110\right)\\ P\left(0|111\right) & P\left(1|111\right) \end{array}\right]=\left[\begin{array}{cc} 1 & 0\\ 0 & 1\\ 1 & 0\\ 1 & 0\\ 1 & 0\\ 1 & 0\\ 1 & 0\\ 1 & 0 \end{array}\right]\;.\;\begin{array}{ccc} 0 & 0 & 0\\ 0 & 0 & 1\\ 0 & 1 & 0\\ 0 & 1 & 1\\ 1 & 0 & 0\\ 1 & 0 & 1\\ 1 & 1 & 0\\ 1 & 1 & 1 \end{array}\\ \;\mathrm{caspase}3=\;0\;1 \end{array}$$

Each element of the above matrix is a conditional transition probability of the form *P*(caspase3|EGF, insulin, TNF). For any given set of 0/1 values for the inputs shown in (6), this gives the probability of the output to be 0 or 1.

### Method to determine the input–output relationships for the pathological caspase3 network

In order to model a pathological molecular channel, one needs to specify the equations that determine the channel output, in terms of the channel inputs. Since the state of each molecule is considered to be binary, i.e., active, 1, or inactive, 0, we need to specify the binary equation of each molecule. The binary equation of each molecule includes all the regulatory inputs to that particular molecule, and symbolically shows how the activity of the molecule is regulated by its different inputs. Based on the physiological mechanisms by which different regulators control the activity of each molecule in the caspase3 network (Figure 1a), we have derived all the binary equations (Table 1).

**Table 1:** **Equations for the caspase3 channel in Figure**1**a**

	**Molecules**	**Equations**
Internal molecules of the channel (listed alphabetically)	1. AKT	AKT = EGFR + insulin
	2. caspase8	caspase8 = cFLIP_L_’ × (ComplexII + ERK)
	3. cFLIP_L_	cFLIP_L_ = NFκB
	4. ComplexI	ComplexI = TNF
	5. ComplexII	ComplexII = TNF + ComplexI
	6. EGFR	EGFR = EGF
	7. ERK	ERK = MEK
	8. IKK	IKK = ComplexI
	9. IRS1	IRS1 = Insulin
	10. JNK1	JNK1 = MKK7
	11. MEK	MEK = EGFR + IRS1
	12. MEKK1ASK1	MEKK1ASK1 = ComplexI
	13. MK2	MK2 = p38
	14. MKK3	MKK3 = MEKK1ASK1
	15. MKK7	MKK7 = MEKK1ASK1
	16. NFκB	NFκB = IKK
	17. p38	p38 = MKK3
Channel output	caspase3	caspase3 = AKT’ × (caspase8 + JNK1 + MK2)

Each equation specifies the input signals to a molecule using the binary operations ’, + and ×, which represent NOT, OR and AND, respectively.

To understand the binary equations (Table 1), we explain three representative equations. Other equations can be similarly explained. The first equation AKT = EGFR + insulin means that the activation of EGFR “or” insulin activates AKT. The last equation caspase3 = AKT’ × (caspase8 + JNK1 + MK2) indicates that the activation of AKT, which is an inhibitor, turns off caspase3, i.e., caspase3 = 0. However, if AKT is inactive (0), activation of caspase8 “or” JNK1 “or” MK2 can activate caspase3. Finally, the third equation cFLIP_L_ = NFκB simply shows that NFκB is an activator for cFLIP_L_.

When all the molecules are functional, the state of the output molecule caspase3 can be determined using the equations (Table 1), for eight different set of inputs. This results in the input–output table (Figure 1b), which is consistent with the experimental findings of Janes *et al.*[20].

On the other hand, when there is dysfunctional molecule in the network, the state of caspase3 can no longer be determined from Figure 1b. Depending on which molecule is dysfunctional, the output state needs to be recalculated for eight different set of inputs, using the network equations (Table 1). For example, when AKT is dysfunctional such that it is always active, 1, irrespective of the states of its inputs EGFR and insulin, caspase3 remains inactive, 0, all the time. The reason is that according to the last equation (Table 1) we have caspase3 = AKT’ × (caspase8 + JNK1 + MK2) = 0 × (caspase8 + JNK1 + MK2) = 0, since AKT = 1 and 1’ = 0 (Additional file 1: Table S2, the row next to AKT = 1). The states of caspase3 when other molecules are dysfunctional are given in Additional file 1: Table S2, for eight different input combinations. Note that a dysfunctional molecule is considered to be either always active, 1, or inactive, 0, and its state remains fixed, no matter what the states of its regulatory inputs are.

### Method for calculating channel transition probabilities in the pathological caspase3 network

Consider the channel model introduced in the paper, where the probability of each molecule to be dysfunctional is *β*, except one dominant molecule such that its dysfunctionality likelihood is *kβ*, *k* ≥ 1. Transformation of a normal molecule to a dysfunctional one is an event that occurs due to mutations or other structural/functional abnormalities. In our model these events are independent. Also only one molecule at a time is considered to be dysfunctional. Since there are seventeen molecules in the channel (Figure 1a), we have 16*β* + *kβ* = 1, which yields *β* = 1/(*k* + 16). So, in our pathological channel model the dysfunctionality likelihoods of the dominant and the other sixteen molecules are *k*/(*k* + 16) and 1/(*k* + 16), respectively. When there is no dominant molecule, i.e., *k* = 1, all the seventeen molecules of the channel are equally likely to be dysfunctional, each with a probability of 1/17.

Each transition probability is the conditional probability *P*(caspase3|EGF, insulin, TNF) that can be calculated using the total probability theorem:

(7) $$\begin{array}{l} P\left(\mathrm{caspase}3|\mathrm{EGF},\mathrm{insulin},\mathrm{TNF}\right)=\sum_{i=1}^{17}\left\{P\left(\mathrm{caspase}3|\mathrm{EGF},\mathrm{insulin},\mathrm{TNF},D_{i}^{0}\right)P\left(D_{i}^{0}\right)+P\left(\mathrm{caspase}3|\mathrm{EGF},\mathrm{insulin},\mathrm{TNF},D_{i}^{1}\right)P\left(D_{i}^{1}\right)\right\}. \end{array}$$

Here $D_{i}^{0}$ represents the event that X_*i*_ is dysfunctional with X_*i*_ = 0, where X_*i*_ is the *i*-th molecule in Table 1. Similarly, $D_{i}^{1}$ denotes the event that X_*i*_ is dysfunctional such that X_*i*_ = 1. It is easy to verify that:

(8) $$P\left(D_{i}^{0}\right)=P\left(X_{i}=0|X_{i}\;\mathrm{is}\;\mathrm{dysfunc}\right)P\left(X_{i}\;\mathrm{is}\;\mathrm{dysfunc}\right)=\left(1/2\right)p_{i}$$

(9) $$P\left(D_{i}^{1}\right)=P\left(X_{i}=1|X_{i}\;\mathrm{is}\;\mathrm{dysfunc}\right)P\left(X_{i}\;\mathrm{is}\;\mathrm{dysfunc}\right)=\left(1/2\right)p_{i}$$

where *p*_*i*_ = *P*(X_*i*_ is dysfunc). Note that in our model a dysfunctional molecule is equally likely to be either 0 or 1. This is why we have *P*(X_*i*_ = 0|X_*i*_ is dysfunc) = *P*(X_*i*_ = 1|X_*i*_ is dysfunc) = 1/2 in (8) and (9). Moreover, *p*_*i*_ = *k*/(*k* + 16) if X_*i*_ is a dominant molecule. Otherwise, *p*_*i*_ = 1/(*k* + 16). The probabilities $P\left(\mathrm{caspase}3|\mathrm{EGF},\mathrm{insulin},\mathrm{TNF},D_{i}^{0}\right)$ and $P\left(\mathrm{caspase}3|\mathrm{EGF},\mathrm{insulin},\mathrm{TNF},D_{i}^{1}\right)$ in (7) are either zero or one, and can be determined using Additional file 1: Table S2. For example, $P\left(1|0,0,1,D_{6}^{0}\right)=1$ since with (EGF, insulin, TNF) = (0, 0, 1) and the dysfunctional EGFR (no. 6 in Additional file 1: Table S2) locked at 0, we have caspase3 = 1. Similarly, one can verify that $P\left(1|0,0,1,D_{6}^{1}\right)=0$. By inserting (8) and (9) into (7) we finally obtain:

(10) $$\begin{array}{l} P\left(\mathrm{caspase}3|\mathrm{EGF},\mathrm{insulin},\mathrm{TNF}\right)=\\ \;\left(1/2\right)\sum_{i=1}^{17}p_{i}\left\{P\left(\mathrm{caspase}3|\mathrm{EGF},\mathrm{insulin},\mathrm{TNF},D_{i}^{0}\right)+P\left(\mathrm{caspase}3|\mathrm{EGF},\mathrm{insulin},\mathrm{TNF},D_{i}^{1}\right)\right\}. \end{array}$$

For the pathological channel model introduced earlier, we have calculated *P*(1|EGF, insulin, TNF) for different input combinations using (10) and Additional file 1: Table S2. They are listed in the second column of the transition probability channel matrix **M** in (11). The first column of **M**, i.e., the conditional probability of caspase3 = 0, is determined according to *P*(0|EGF, insulin, TNF) = 1 − *P*(1|EGF, insulin, TNF):

(11) $$M_{\mathrm{abnormal}\;\mathrm{channel}}=\left[\begin{array}{cc} P\left(0|000\right) & P\left(1|000\right)\\ P\left(0|001\right) & P\left(1|001\right)\\ P\left(0|010\right) & P\left(1|010\right)\\ P\left(0|011\right) & P\left(1|011\right)\\ P\left(0|100\right) & P\left(1|100\right)\\ P\left(0|101\right) & P\left(1|101\right)\\ P\left(0|110\right) & P\left(1|110\right)\\ P\left(0|111\right) & P\left(1|111\right) \end{array}\right]=\left[\begin{array}{cc} 1-P\left(1|000\right) & \frac{1}{2}\left(p_{2}+p_{4}+p_{5}+p_{7}+\sum_{i=9}^{15}p_{i}+p_{17}\right)\\ 1-P\left(1|001\right) & \frac{1}{2}\left(p_{1}+2\sum_{i=2}^{5}p_{i}+p_{6}+2\sum_{i=7}^{11}p_{i}+p_{12}+2\sum_{i=13}^{17}p_{i}\right)\\ 1-P\left(1|010\right) & p_{1}/2\\ 1-P\left(1|011\right) & p_{1}/2\\ 1-P\left(1|100\right) & p_{1}/2\\ 1-P\left(1|101\right) & \left(p_{1}+p_{6}\right)/2\\ 1-P\left(1|110\right) & p_{1}/2\\ 1-P\left(1|111\right) & p_{1}/2 \end{array}\right].$$

When a molecule is dominant, its *p*_*i*_ becomes *k* times greater than the other *p*_*i*_ s in our model. Depending on which molecule is dominant, **M** in (11) takes a different form. More specifically, the seventeen molecules of the channel (Figure 1a) can be categorized into five classes: {1}, {6}, {12}, {3,8,16}, and {2,4,5,7,9,10,11,13,14,15,17}, where numbers refer to the molecules in Table 1. In what follows, we express **M** in terms of the dominance factor *k* for these five set of molecules:

(12) $$M_{\mathrm{abnormal}\;\mathrm{channel}}=\frac{1}{2k+32}\left[\begin{array}{cc} 2k+20 & 12\\ k+2 & k+30\\ k+32 & k\\ k+32 & k\\ k+32 & k\\ k+31 & k+1\\ k+32 & k\\ k+32 & k \end{array}\right]\left(\mathrm{AKT}\;\mathrm{is}\;\mathrm{dominant}\right),$$

(13) $$M_{\mathrm{abnormal}\;\mathrm{channel}}=\frac{1}{2k+32}\left[\begin{array}{cc} 2k+20 & 12\\ k+2 & k+30\\ 2k+31 & 1\\ 2k+31 & 1\\ 2k+31 & 1\\ k+31 & k+1\\ 2k+31 & 1\\ 2k+31 & 1 \end{array}\right]\left(\mathrm{EGFR}\;\mathrm{is}\;\mathrm{dominant}\right),$$

(14) $$M_{\mathrm{abnormal}\;\mathrm{channel}}=\frac{1}{2k+32}\left[\begin{array}{cc} k+21 & k+11\\ k+2 & k+30\\ 2k+31 & 1\\ 2k+31 & 1\\ 2k+31 & 1\\ 2k+30 & 2\\ 2k+31 & 1\\ 2k+31 & 1 \end{array}\right]\left(\mathrm{MEKK}1\mathrm{ASK}1\;\mathrm{is}\;\mathrm{dominant}\right),$$

(15) $$M_{\mathrm{abnormal}\;\mathrm{channel}}=\frac{1}{2k+32}\left[\begin{array}{cc} 2k+20 & 12\\ 3 & 2k+29\\ 2k+31 & 1\\ 2k+31 & 1\\ 2k+31 & 1\\ 2k+30 & 2\\ 2k+31 & 1\\ 2k+31 & 1 \end{array}\right]\left({\mathrm{cFLIP}}_{L}\mathrm{or}\;\mathrm{IKK}\;\mathrm{or}\;\mathrm{NF\kappa B}\;\mathrm{is}\;\mathrm{dominant}\right),$$

(16) $$M_{\mathrm{abnormal}\;\mathrm{channel}}=\frac{1}{2k+32}\left[\begin{array}{cc} k+21 & k+11\\ 3 & 2k+29\\ 2k+31 & 1\\ 2k+31 & 1\\ 2k+31 & 1\\ 2k+30 & 2\\ 2k+31 & 1\\ 2k+31 & 1 \end{array}\right]\;\begin{array}{c} (\mathrm{caspase}8\;\mathrm{or}\;\mathrm{ComplexI}\;\mathrm{or}\;\mathrm{ComplexII}\;\mathrm{or}\;\mathrm{ERK}\\ \mathrm{or}\;\mathrm{IRS}1\;\mathrm{or}\;\mathrm{JNK}1\;\mathrm{or}\;\mathrm{MEK}\;\mathrm{or}\;\mathrm{MK}2\;\mathrm{or}\;\mathrm{MKK}3\;\\ \mathrm{or}\;\mathrm{MKK}7\;\mathrm{or}\;p38\;\mathrm{is}\;\mathrm{dominant}), \end{array}$$

Note that for *k* = 1, all the **M** matrices in (12)-(16) reduce to the following matrix in (17). The associated channel transition probability diagram is shown in Figure 1d. It corresponds to the pathological channel model where all the molecules are equally probable to be dysfunctional.

(17) $$M_{\mathrm{abnormal}\;\mathrm{channel}}^{k=1}=\left[\begin{array}{cc} 22/34 & 12/34\\ 3/34 & 31/34\\ 33/34 & 1/34\\ 33/34 & 1/34\\ 33/34 & 1/34\\ 32/34 & 2/34\\ 33/34 & 1/34\\ 33/34 & 1/34 \end{array}\right]\;\left(\mathrm{all}\;\mathrm{molecules}\;\mathrm{are}\;\mathrm{equally}\;\mathrm{likely}\;\mathrm{to}\;\mathrm{be}\;\mathrm{dysfunctional}\right),$$

### Method for calculating the transmission error probability *P*_*e*_

According to the channel transition probability diagram (Figure 1c), a transmission error occurs when the inputs are (EGF, insulin, TNF) = (0, 0, 1) but the output is caspase3 = 0. Additionally, if the inputs are anything but (EGF, insulin, TNF) = (0, 0, 1), caspase3 = 1 is an incorrect output and indicates transmission error as well. Therefore, using the total probability theorem, the transmission error probability can be written as:

(18) $$P_{e}=\sum P\left(\mathrm{incorrect}\;\mathrm{output}|\mathrm{EGF},\;\mathrm{insulin},\;\mathrm{TNF}\right)P\left(\mathrm{EGF},\;\mathrm{insulin},\;\mathrm{TNF}\right)$$

Since in our model all the eight input combinations are equally probable, (18) can be simplified to:

(19) $$\begin{array}{l} P_{e}=\frac{1}{8}\{P\left(0|001\right)\\ +P\left(1|000\right)+P\left(1|010\right)+P\left(1|011\right)+P\left(1|100\right)+P\left(1|101\right)+P\left(1|110\right)+P\left(1|111\right)\}. \end{array}$$

The conditional probabilities in (19) can be easily obtained from the matrices in (12)-(16). This results in the following expressions for *P*_*e*_ versus *k*, depending on which molecule is dominant:

(20) $$P_{e}=\frac{7k+15}{16\left(k+16\right)}\;\left(\mathrm{AKT}\;\mathrm{is}\;\mathrm{dominant}\right),$$

(21) $$P_{e}=\frac{2k+20}{16\left(k+16\right)}\;\left(\mathrm{EGFR}\;\mathrm{or}\;\mathrm{MEKK}1\mathrm{ASK}1\;\mathrm{is}\;\mathrm{dominant}\right),$$

(22) $$P_{e}=\frac{22}{16\left(k+16\right)}\;\left({\mathrm{cFLIP}}_{L}\mathrm{or}\;\mathrm{IKK}\;\mathrm{or}\;\mathrm{NF\kappa B}\;\mathrm{is}\;\mathrm{dominant}\right),$$

(23) $$P_{e}=\frac{k+21}{16\left(k+16\right)}\;\begin{array}{c} (\mathrm{caspase}8\;\mathrm{or}\;\mathrm{ComplexI}\;\mathrm{or}\;\mathrm{ComplexII}\mathrm{or}\;\mathrm{ERK}\\ \mathrm{or}\;\mathrm{IRS}1\;\mathrm{or}\;\mathrm{JNK}1\mathrm{or}\;\mathrm{MEK}\mathrm{or}\;\mathrm{MK}2\;\mathrm{or}\;\mathrm{MKK}3\\ \mathrm{or}\;\mathrm{MKK}7\;\mathrm{or}\;p38\;\mathrm{is}\;\mathrm{dominant}). \end{array}$$

When *k* = 1, all the above equations reduce to *P*_*e*_ = 11/136, as mentioned in the paper. This is the case where all the molecules are equally likely to be dysfunctional. Plots of the *P*_*e*_ equations in (20)-(23) versus *k* are provided in Figure 2a. It is instructive to look at the transmission error probability as *k* approaches infinity, i.e., only one molecule is dysfunctional in the channel with probability one and the chance of others to be dysfunctional is zero. As *k* → ∞, *P*_*e*_ in equations (20)-(23) reduces to 7/16, 2/16, 0 and 1/16, respectively. These are consistent with what said in the paper, i.e., AKT plays a significant role in this molecular network. This means that when AKT is dysfunctional while the other molecules are functional, there is a high probability that the input message from ligands will not be correctly delivered to caspase3. On the other hand, cFLIP_L_ and IKK and NFκB introduce zero transmission error, when one of them is the only dysfunctional molecule. The remaining molecules provides a relatively small *P*_*e*_, i.e., 2/16 and 1/16.

### Method for calculating the signaling capacity *C*

To calculate the capacity of the molecular channel (Figure 1a), one needs the transition probability channel matrix **M**. Depending on which molecule is dominant in the pathological channel, one can use the matrices given in (12)-(16). The Arimoto algorithm is used to numerically calculate the signaling capacity *C* (Figure 2b).

### Methods for calculating the transmission error probability *P*_*e*_ and signaling capacity *C* in the pathological T cell network

Binary equations of the T cell network [24] that represent how it functions in response to different inputs are provided in Additional file 1: Table S1, using the notation of this paper. Following the same methods used for the pathological casepase3 network, one can calculate *P*_*e*_ and *C* in the pathological T cell channel, using the logic equations (Additional file 1: Table S1).

## Competing interests

The authors declare that they have no competing interests.

## Authors’ contributions

AA and EE conceived the ideas. IH and AA did the computational and engineering research, and EE did the molecular biology research. They all wrote the paper. All authors read and approved the final manuscript.

## Additional file

## Supplementary Material

Additional file 1: **Quantitative Analysis of Intracellular Communication and Signaling Errors in Signaling Networks**[1],[6],[7],[20]–[24],[34]–[36]. Click here for file
