# Supplementary material for: Quantitative analysis of intracellular communication and signaling errors in signaling networks
Source: BMC Syst Biol. 2014 Aug 21;8:89. doi: 10.1186/s12918-014-0089-z (PMC4255782; doi:10.1186/s12918-014-0089-z)
Supplement: Additional file 1: — Quantitative Analysis of Intracellular Communication and Signaling Errors in Signaling Networks[1],[6],[7],[20]–[24],[34]–[36]. [file s12918-014-0089-z-S1.pdf]

Supplementary Information

Quantitative Analysis of Intracellular Communication and Signaling Errors in Signaling Networks

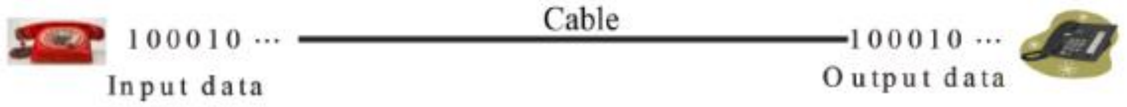

a

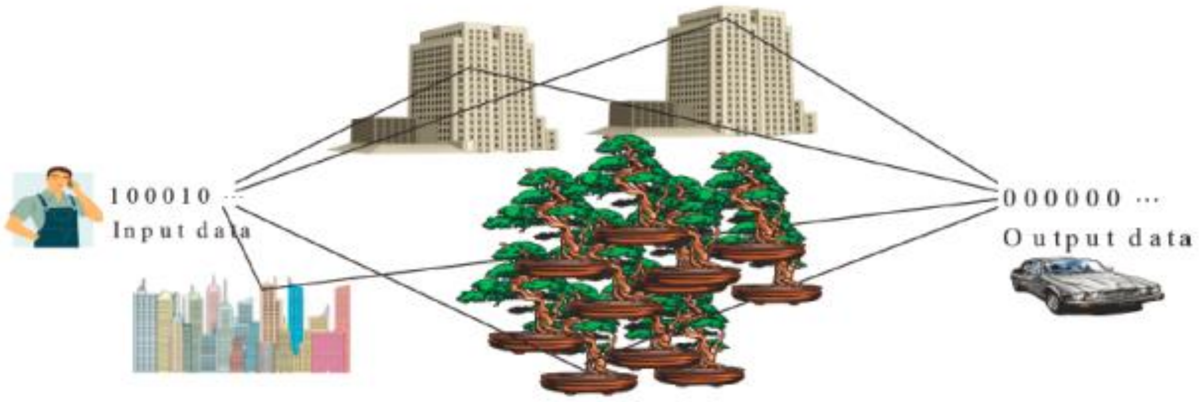

b

Figure S1 (a) An error-free communication channel with no transmission error. **(b)** An erroneous communication channel where transmitted 1’s are erroneously received as 0’s by the receiver.

Signal Transmission in a Toy Pathway

As depicted by the example (Figure S1a), digital communication in an error-free channel does not face any transmission error. A general block diagram representation of an error-free channel is shown in Figure S2a, where for example, the digital input sequence 100010 is transferred to the output without any error. To explain this concept in a molecular biology context, consider the simple toy pathway (Figure S2b). Complex real signaling networks with many molecules and interactions are fully studied in the paper afterwards. This toy pathway is introduced just to explain the basic ideas and concepts using a simple example, and the developed methods are applicable to large signaling networks with multiple inputs and outputs as well, as shown later in the paper. The toy pathway consists of four molecules *a*, *b*, *c* and *d*, where *a* and *d* are a ligand and a transcription factor, respectively, and *b* and *c* are two intermediate molecules. This pathway can resemble a number of MAPK signaling cascades [1]. Presence of ligand *a* results in the activation of molecules *b*, *c* and subsequently *d*. From a communication engineering perspective, we consider the cascade *b* and *c* as a communication channel, where *a* and *d* are the input and output of the channel, respectively. Using the digital communication terminology, active and inactive states of a molecule can be represented by binary digits 1 and 0, respectively. Therefore, presence of ligand *a* can be written as $a=1$, i.e., the binary digit 1 is applied to the channel input, to be transmitted to the output. In a normal pathway, this results in the activation *b* and *c*, i.e., $b=1$ and $c=1$, and subsequently the activation of *d*, represented by $d=1$. We can see that in this normal pathway, a 1 at the channel input is successfully transmitted to the output. On the other hand, when ligand a is not present, $a=0$, other molecules remain inactive, i.e., $b=0$, $c=0$, and $d=0$. Again we see in this normal pathway, the binary digit 0 applied to the channel input is correctly delivered to the destination, the channel output. The sequence $\{1,0,0,0,1,0, ...\}$ at the channel input (Figure S2a) corresponds to {ligand is

present, no ligand, no ligand, no ligand, ligand is present, no ligand, …} whereas the output sequence $\{1, 0, 0, 0, 1, 0, ...\}$ (Figure S2a) implies {*d* is active, *d* is inactive, *d* is inactive, *d* is inactive, *d* is active, *d* is inactive, …}. The channel transition probability diagram (Figure S2c) visually shows these error-free transmissions. The upper branch shows that a 0 at the channel input appears at the channel output with probability 1. Similarly, the lower branch shows that there is a 100% chance for a 1 at the input to successfully travel through the channel and reach the output.

In an erroneous communication channel, however, there might be some transmission errors (Figure S2d). For example, all the 0's are transmitted correctly, whereas all the 1's are incorrectly received as 0's (Figure S2d). A biological interpretation for this example can be provided using the toy pathway, when one of the molecules in the channel, say *c*, is dysfunctional (Figure S2e) due to some mutations or other structural/functional abnormalities. This is symbolically shown by a broken circle (Figure S2e). This dysfunctional molecule cannot respond properly to its input signal from molecule *b*, when *b* is activated due to ligand binding. This means that *d* will not be activated, due to the pathologic channel behavior (dysfunctional molecule *c* in this example). In the language of digital communication, a 1 at the channel input (presence of ligand *a*) incorrectly appears as a 0 at the channel output (inactivity of the transcription factor *d*). There are two transmission errors in our example (Figure S2d), which correspond to two incidents of ligand binding which have failed to activate the transcription factor *d*. Transmission error created by this channel is symbolically shown in the channel transition probability diagram (Figure S2f), via an arrow from 1 at the input to 0 at the output. The 1 above the arrow indicates that with a 100% probability, a 1 at the input appears as a 0 at the output.

To quantify signal transmission in a signal transduction pathway, one can calculate the transmission error probability P_e. As a simple scenario, we consider 50% of the inputs to be 1, which are all transmitted incorrectly, whereas the rest are 0, which are transmitted without any error. In this example, for the functional (Figure S2b) and dysfunctional (Figure S2e) toy pathways, we have calculated that $P_e = 0$ and $P_e = 0.5$, respectively, assuming there is an equal probability of 0.5 to have a 0 or 1 at the input (see Supplementary Methods). Thus, there is no chance of signal transmission error in the normal pathway, whereas there is a 50% chance of having an incorrect output in the abnormal pathway (no activation of the output transcription factor, although a ligand is bound).

Signal Transmission and Signaling Capacity of the Toy Pathway

The concepts of signal transmission in signaling networks and signal loss in abnormal (dysfunctional) networks can be quantified using entropy, equivocation and mutual information [6,7]. The concept of mutual information has been used in other biology contexts, for example, to understand the genetic basis of E. coli tolerance to ethanol [34], identify adaptive mutations [35] and study other genomic and evolution-related problems [36]. The goal of the present paper, however, is different from those studies. More specifically, here the goal is to develop a systematic communication/signaling framework to quantify and analyze intracellular communication and signaling among molecules in a cell. This approach allows to find critical molecules whose dysfunction causes significant signaling errors from ligands or secondary messengers to transcription factors. Signal transduction errors result in the deregulation of some output molecules such as transcription factors, which detrimentally affect some important cellular functions and ultimately contribute to the development of the pathology.

The amount of signal provided to the input of a pathway (Figure S2b and Figure S2e) is the source or input *entropy* $H(a)$. When the presence or absence of the ligand a at the input are

equally likely, one can show $H(a)=1$ bit (see Supplementary Methods). Here bit is the unit of information and is an acronym for binary unit. *Equivocation* $H(a\,|\,d)$ can be considered a measure of the signal loss in a signaling pathway whose input and output are *a* and *d*, respectively. In the normal toy pathway (Figure S2b) we have $H(a\,|\,d)=0$ (see Supplementary Methods). This means there is no signal loss in the normal pathway, as there is no dysfunctional molecule there. On the other hand, the amount of signal lost in the abnormal toy pathway (Figure S2e) is $H(a\,|\,d)=1$ bit (see Supplementary Methods). This is because of the dysfunctional molecule (Figure S2e) that makes the channel abnormal, which results in the loss of signal provided to the signaling pathway by a ligand. The amount of signal transferred through the molecular signaling channel from the input ligand *a* to the output transcription factor *d* can be measured by the *mutual information* $I(a;d)$. The relationship between $I(a;d)$, input entropy $H(a)$ and equivocation $H(a\,|\,d)$ in the toy pathway (Figure S2b and Figure S2e) is given by $I(a;d)=H(a)-H(a\,|\,d)$. This means the amount of transferred signal from the input ligand *a* to the output transcription factor *d* through the signaling pathway is equal to the amount of signal provided to the pathway input via the ligand *a*, minus the signal that might be lost in the pathway due to some dysfunctional molecules. In the normal pathway (Figure S2b) we showed $H(a)=1$ and $H(a\,|\,d)=0$, which results in $I(a;d)=1-0=1$ bit/transmission. This means that all the signal provided to the input of the normal pathway is successfully conveyed to the output and no signal is lost. On the other hand, in the abnormal pathway (Figure S2e) our calculations provided $H(a)=1$ and $H(a\,|\,d)=1$, which results in $I(a;d)=1-1=0$ bit/transmission. This is biologically relevant and implies that the signal provided to the input of the abnormal pathway is lost in the channel, and not provided to the output. That is why the output transcription factor *d* remains to be inactive when a molecule in the pathway is dysfunctional, irrespective of the signals applied to the pathway input.

The maximum signal content that can be transmitted through a communication channel is called the *capacity C* [6,7]. In our toy pathways (Figure S2b and Figure S2e) it can be written as $C = \max I(a;d)$, and represents the maximum amount of signal that can be transmitted from the ligand *a* to the transcription factor *d*. The maximization needs to be done over all possible probability distributions of the input. It can be shown that for the normal and abnormal toy pathways we have $C = 1$ and $C = 0$ bit/transmission, respectively (see Supplementary Methods). So, the maximum amount of signal that the pathway can convey reduces, if there is an abnormality in the channel, e.g., a dysfunctional molecule.

The concept of capacity plays a key role in communication systems and signal transmission [6,7]. In the molecular biology context, if due to the presence of some dysfunctional molecules the signaling network capacity is significantly reduced, then according to communication and signal transmission theorems, it will not be possible for the cell to successfully convey the signal to the target protein via the network. This failure of the cell to properly regulate a target protein may result in a transition from the normal state to the disease state. In order to show the application of signaling capacity in real biological networks, in the paper we first have developed a communication channel model for the caspase3 signaling network [20,21,22]. This has allowed to quantitatively show how dysfunctional molecules can affect the transmission error probability and the capacity of an important signaling network known to be involved in several pathological conditions [20,21,22]. Furthermore, in the paper we have analyzed several experimental measurements of different molecules involved in caspase-3 signaling networks [20] and found supporting biological evidence that indicated the developed method using signals and communication systems concepts can be verified experimentally. We have then applied the proposed method to a large T cell signaling network [24]. As explained in the paper, the results

obtained using this novel methodology are in agreement with the experimental data. This demonstrates the biological relevance of the findings of the method in pathological networks.

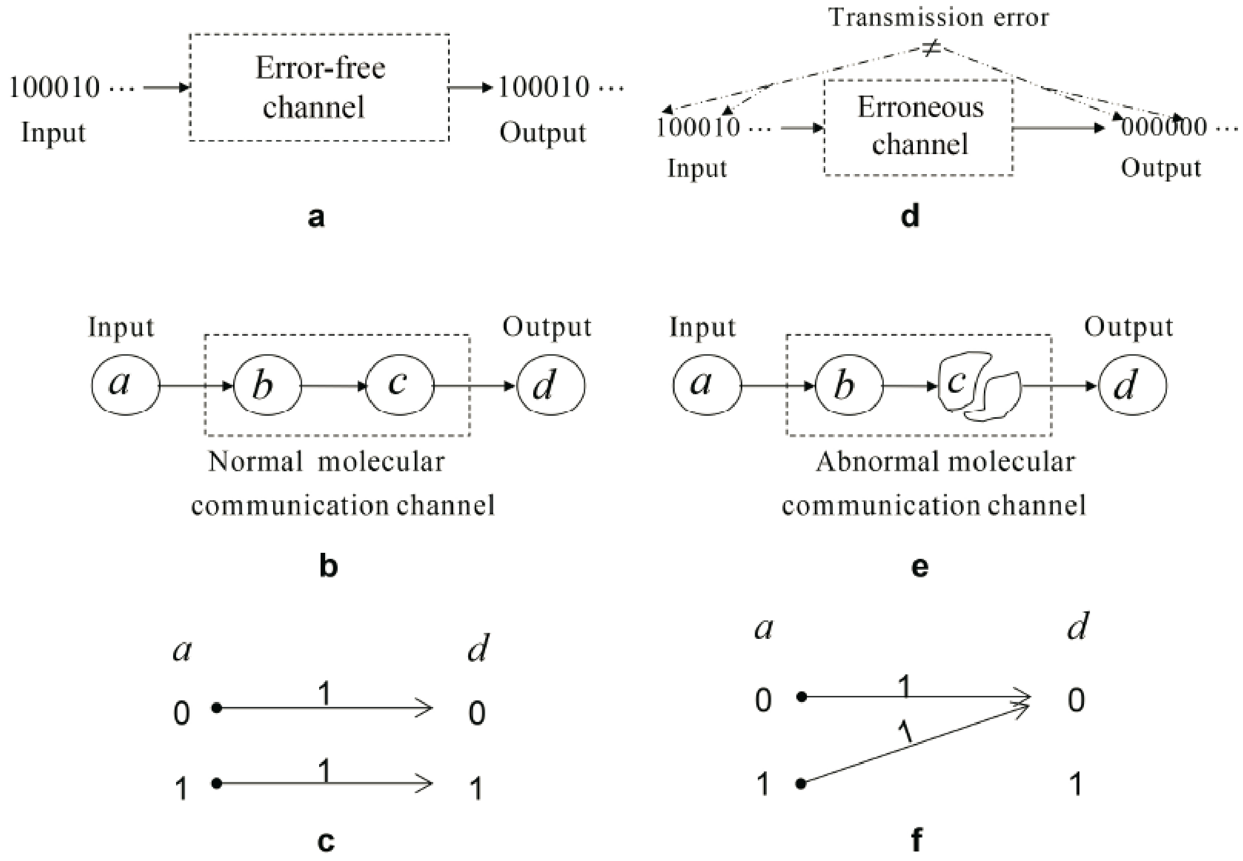

Figure S2 (a) Schematic representation of an error-free communication channel through which binary signals, 0 and 1, can be transmitted without any transmission error. **(b)** A toy signaling pathway which consists of four molecules *a*, *b*, *c* and *d*, where *a* and *d* are a ligand and a transcription factor, respectively, whereas *b* and *c* are two intermediate molecules. Presence of ligand *a* results in the activation of molecules *b*, *c* and subsequently *d*. **(c)** Transition probability diagram of the normal channel, where numbers above the arrows are transition probabilities. **(d)** Schematic representation of an erroneous communication channel, where 1's are incorrectly delivered to the output. **(e)** The toy pathway where molecule *c* in the channel is dysfunctional (symbolically shown by a broken circle). This might be because of mutations or some structural/functional abnormalities. This dysfunctional molecule cannot respond properly to its

input signal from molecule *b*, and therefore cannot regulate molecule *d*. **(f)** Transition probability diagram of the abnormal (pathological) channel, where numbers above the arrows are transition probabilities.

Supplementary Method for calculating transmission error probability in the toy pathway.

The input and output of the toy pathway (Figure S2b and Figure S2e) are molecules *a* and *d*, respectively. Transmission error probability P_e can be calculated using the total probability theorem [23]:

$$P_e = P(a=0)P(d=1 \mid a=0) + P(a=1)P(d=0 \mid a=1), \qquad \text{(S1)}$$

where $P(a=0)$ and $P(a=1)$ are the probabilities of *a* to be 0 or 1, respectively. The conditional probability $P(d=1 \mid a=0)$ specifies the likelihood of the output *d* to be 1 (active), if the input *a* is 0 (inactive). The conditional probability $P(d=0 \mid a=1)$ is similarly defined. According to the channel transition probability diagram of the normal pathway (Figure S2c) we have $P(d=1 \mid a=0)=0$, as no arrow connects 0 at the input to 1 at the output. It can be similarly shown that $P(d=0 \mid a=1)=0$. By substituting these results into (S1) we obtain $P_e=0$ for the normal pathway.

Based on the channel transition probability diagram of the abnormal (pathological) pathway Figure S2f) we still have $P(d=1 \mid a=0)=0$, as no arrow connects 0 at the input to 1 at the output. However, due to the arrow connecting 1 at the input to 0 at the output with probability 1, we obtain $P(d=0 \mid a=1)=1$. By substituting these results into (S1) we obtain $P_e=P(a=1)$ for the abnormal pathway. This makes sense because whenever there is a 1 at the input, it will be incorrectly transmitted to the output. So, transmission error probability in this abnormal pathway is the same as the probability of having 1 at the input, i.e., $P(a=1)$. Here we consider the case where there is an equal probability of 1/2 to have a 0 or 1 at the input, i.e., $P(a=0)=P(a=1)=1/2$. This results in $P_e=1/2$ for the abnormal pathway.

Supplementary Methods for calculating the input entropy, equivocation and signaling capacity in the toy pathway. Entropy [6,7] of the input *a* (Figure S2b and Figure S2e) can be calculated according to:

$$H(a) = -P(a=0)\log_2 P(a=0) - P(a=1)\log_2 P(a=1)\,, \qquad \text{(S2)}$$

where $\log_2(.)$ is the base-2 logarithm. When there is an equal probability of 1/2 to have a 0 or 1 at the input, i.e., $P(a=0) = P(a=1) = 1/2$, then it is easy to verify $H(a) = 1$. This is because $\log_2(1/2) = -1$.

Equivocation [6,7] for the pathway with input *a* and output *d* (Figure S2b and Figure S2e) can be calculated according to:

$$\begin{aligned} H(a \mid d) = &-P(a=0,d=0)\log_2 P(a=0 \mid d=0) \\ &- P(a=0,d=1)\log_2 P(a=0 \mid d=1) \\ &- P(a=1,d=0)\log_2 P(a=1 \mid d=0) \\ &- P(a=1,d=1)\log_2 P(a=1 \mid d=1). \end{aligned} \qquad \text{(S3)}$$

Based on the relationship $P(a,d) = P(a \mid d)P(d) = P(d \mid a)P(a)$ between joint and conditional probabilities, (S3) can be re-written as:

$$\begin{aligned} H(a \mid d) = &-P(d=0 \mid a=0)P(a=0)\log_2\{P(d=0 \mid a=0)P(a=0)/P(d=0)\} \\ &- P(d=1 \mid a=0)P(a=0)\log_2\{P(d=1 \mid a=0)P(a=0)/P(d=1)\} \\ &- P(d=0 \mid a=1)P(a=1)\log_2\{P(d=0 \mid a=1)P(a=1)/P(d=0)\} \\ &- P(d=1 \mid a=1)P(a=1)\log_2\{P(d=1 \mid a=1)P(a=1)/P(d=1)\}, \end{aligned} \qquad \text{(S4)}$$

where the output probabilities are given by:

$$\begin{aligned} P(d=0) &= P(a=0)P(d=0 \mid a=0) + P(a=1)P(d=0 \mid a=1), \\ P(d=1) &= P(a=0)P(d=1 \mid a=0) + P(a=1)P(d=1 \mid a=1). \end{aligned} \qquad \text{(S5)}$$

By substituting the conditional probabilities $P(d \mid a)$ in (S4) and (S5) with the their numerical values presented in the channel transition probability diagrams (Figure S2c and Figure S2f), one can easily calculate $H(a \mid d)$. It is straightforward to verify that $H(a \mid d) = 0$ in the normal pathway (Figure S2b), since $\log_2(1) = 0$. This means no signal loss in the normal pathway. For the abnormal pathway (Figure S2e) we obtain $H(a \mid d) = H(a)$. When there is an equal

probability of 1/2 to have a 0 or 1 at the input, i.e., $P(a=0)=P(a=1)=1/2$, this reduces to $H(a|d)=1$.

To calculate the capacity of normal and abnormal pathways (Figure S2b and Figure S2e), we note that:

$$I(a;d)=H(a)-H(a|d)=H(a)-0=H(a)\,,\quad \text{normal pathway,} \tag{S6}$$

$$I(a;d)=H(a)-H(a|d)=H(a)-H(a)=0\,,\quad \text{abnormal pathway.} \tag{S7}$$

Using (S2) and by substituting $P(a=1)=1-P(a=0)$ there, (6) can be written as:

$$I(a;d)=-P(a=0)\log_2 P(a=0)-\{1-P(a=0)\}\log_2\{1-P(a=0)\}\,,\quad \text{normal pathway.} \tag{S8}$$

To find the capacity of the normal pathway, the maximum of $I(a;d)$ in (S8) needs to be found. The plot of $I(a;d)$ in (S8) versus $P(a=0)$ looks like a horseshoe, which has a maximum of 1 at $P(a=0)=1/2$. Therefore in the normal pathway we have $C=1$. In the abnormal pathway we simply obtain $C=0$, since according to (S7), the mutual information in the abnormal pathway is zero.

Molecules	Binary equations
abl(t)	abl(t)=lckp1(t)|fyn(t)
akap79	akap79=0
ap1(t)	ap1(t)=fos(t)&jun(t)
bad(t)	bad(t)=~pkb(t)
bcat(t)	bcat(t)=~gsk3(t)
bcl10	bcl10=1
bclxl(t)	bclxl(t)=~bad(t)
ca(t)	ca(t)=ip3(t)
cabin1(t)	cabin1(t)=~camk4(t)
calcin(t)	calcin(t)=(~cabin1(t))&(~akap79)&(~calpr1)&cam(t)
calpr1	calpr1=0
cam(t)	cam(t)=ca(t)
camk2(t)	camk2(t)=cam(t)
camk4(t)	camk4(t)=cam(t)
card11	card11=1
card11a(t)	card11a(t)=card11&bcl10&malt1
cblb(t+1)	cblb(t+1)=~cd28
ccblp1(t+1)	ccblp1(t+1)=zap70(t)
ccblp2(t+1)	ccblp2(t+1)=fyn(t)
cd28	Input node
cd4	Input node
cd45	cd45=1
cdc42	cdc42=0
cre(t)	cre(t)=creb(t)
creb(t)	creb(t)=rsk(t)
csk(t)	csk(t)=pag(t)
cyc1(t)	cyc1(t)=~gsk3(t)
dag(t)	dag(t)=(~dgk(t))&plcga(t)
dgk(t+1)	dgk(t+1)=tcrb(t)
erk(t)	erk(t)=mek(t)
fkhr(t)	fkhr(t)=~pkb(t)
fos(t)	fos(t)=erk(t)
fyn(t)	fyn(t)=tcrb(t)|(lckp1(t)&cd45)
gab2(t+1)	gab2(t+1)=lat(t)&zap70(t)&(gads(t)|grb2(t))
gadd45	gadd45=1
gads(t)	gads(t)=lat(t)
gap	gap=0
grb2(t)	grb2(t)=lat(t)
gsk3(t)	gsk3(t)=~pkb(t)
hpk1(t)	hpk1(t)=lat(t)
ikb(t)	ikb(t)=~ikkab(t)
ikkab(t)	ikkab(t)=ikkg(t)&camk2(t)
ikkg(t)	ikkg(t)=pkcth(t)&card11a(t)
ip3(t)	ip3(t)=plcga(t)
itk(t)	itk(t)=slp76(t)&zap70(t)&pip3(t)
jnk(t)	jnk(t)=mekk1(t)|mkk4(t)
jun(t)	jun(t)=jnk(t)
lat(t)	lat(t)=zap70(t)
lckp1(t)	lckp1(t)=(~shp1(t))&(~csk(t))&cd45&cd4
lckp2(t)	lckp2(t)=tcrb(t)
malt1	malt1=1
mek(t)	mek(t)=raf(t)

mekk1(t)	mekk1(t)=hpk1(t)|cdc42|rac1p2(t)
mkk4(t)	mkk4(t)=mlk3(t)|mekk1(t)
mlk3(t)	mlk3(t)=hpk1(t)|rac1p1(t)
nfat(t)	nfat(t)=calcin(t)
nfkb(t)	nfkb(t)=~ikb(t)
p21c(t)	p21c(t)=~pkb(t)
p27k(t)	p27k(t)=~pkb(t)
p38(t)	p38(t)=((~gadd45)&zap70(t))|mekk1(t)
p70s(t)	P70s(t)=pdk1(t)
pag(t)	pag(t)=~tcrb(t)
pag(t+1)	pag(t+1)=fyn(t)
pdk1(t)	pdk1(t)=pip3(t)
pi3k(t)	pi3k(t)=((~cblb(t))&x(t))|((~cblb(t))&lckp2(t))
pip3(t)	pip3(t)=pi3k(t)&(~ship1)&(~pten)
pkb(t)	pkb(t)=pdk1(t)
pkcth(t)	pkcth(t)=pdk1(t)&dag(t)&vav1(t)
plcga(t)	plcga(t)=plcgb(t)&(~ccblp2(t))&slp76(t)&zap70(t)& vav1(t)&(itk(t)|rlk(t))
plcgb(t)	plcgb(t)=lat(t)
pten	pten=0
rac1p1(t)	rac1p1(t)=vav1(t)
rac1p2(t)	rac1p2(t)=vav3(t)
raf(t)	raf(t)=ras(t)
ras(t)	ras(t)=(~gap)&rasgrp(t)&sos(t)
rasgrp(t)	rasgrp(t)=dag(t)
rlk(t)	rlk(t)=lckp1(t)
rsk(t)	rsk(t)=erk(t)
sh3bp2(t)	sh3bp2(t)=zap70(t)&lat(t)
ship1	ship1=0
shp1(t+1)	shp1(t+1)=(~erk(t))&lckp1(t)
shp2(t)	shp2(t)=gab2(t)
slp76(t)	slp76(t)=(~gab2(t))&zap70(t)&gads(t)
sos(t)	sos(t)=grb2(t)
sre(t)	sre(t)=rac1p2(t)|cdc42
tcrb(t)	tcrb(t)=(~ccblp1(t))&tcrlig
tcrlig	Input node
tcrp(t)	tcrp(t)=(tcrb(t)&lckp1(t))|(tcrb(t)&fyn(t))
vav1(t)	vav1(t)=(sh3bp2(t)&zap70(t))|x(t)
vav3(t)	vav3(t)=sh3bp2(t)
x(t)	x(t)=cd28
zap70(t)	zap70(t)=(~ccblp1(t))&abl(t)&tcrp(t)

Table S1 Binary equations for the T cell channel in Figure 4a. Each binary equation specifies the input signals to a molecule using the binary operations ~, | and &, which represent NOT, OR and AND, respectively. The symbol t represents the current time whereas t+1 symbolically stands for the next time interval.

Dysfunctional internal molecules of the channel (listed alphabetically)	Channel input molecules: EGF, insulin, TNF	0,0,0	0,0,1	0,1,0	0,1,1	1,0,0	1,0,1	1,1,0	1,1,1
	a. AKT=0	0	1	1	1	1	1	1	1
	AKT=1	0	0	0	0	0	0	0	0
	b. caspase8=0	0	1	0	0	0	0	0	0
	caspase8=1	1	1	0	0	0	0	0	0
	c. cFLIPL=0	0	1	0	0	0	0	0	0
	cFLIPL=1	0	1	0	0	0	0	0	0
	d. ComplexI=0	0	1	0	0	0	0	0	0
	ComplexI=1	1	1	0	0	0	0	0	0
	e. ComplexII=0	0	1	0	0	0	0	0	0
	ComplexII=1	1	1	0	0	0	0	0	0
	f. EGFR=0	0	1	0	0	0	1	0	0
	EGFR=1	0	0	0	0	0	0	0	0
	g. ERK=0	0	1	0	0	0	0	0	0
	ERK=1	1	1	0	0	0	0	0	0
	h. IKK=0	0	1	0	0	0	0	0	0
	IKK=1	0	1	0	0	0	0	0	0
	i. IRS1=0	0	1	0	0	0	0	0	0
	IRS1=1	1	1	0	0	0	0	0	0
	j. JNK1=0	0	1	0	0	0	0	0	0
	JNK1=1	1	1	0	0	0	0	0	0
	k. MEK=0	0	1	0	0	0	0	0	0
	MEK=1	1	1	0	0	0	0	0	0
	l. MEKK1ASK1=0	0	0	0	0	0	0	0	0
	MEKK1ASK1=1	1	1	0	0	0	0	0	0
	m. MK2=0	0	1	0	0	0	0	0	0
	MK2=1	1	1	0	0	0	0	0	0
	n. MKK3=0	0	1	0	0	0	0	0	0
	MKK3=1	1	1	0	0	0	0	0	0
	o. MKK7=0	0	1	0	0	0	0	0	0
	MKK7=1	1	1	0	0	0	0	0	0
	p. NFkappaB=0	0	1	0	0	0	0	0	0
	NFkappaB=1	0	1	0	0	0	0	0	0
	q. p38=0	0	1	0	0	0	0	0	0
	p38=1	1	1	0	0	0	0	0	0

Table S2 The state of the caspase3 channel output (Figure 1a), for eight different input states, when a molecule in the channel is dysfunctional. The column headers represent eight different input states. The row headers specify the dysfunctional molecule and its fixed state.

List of molecules in the T cell network (Figure 4a) for which transmission error probability is 0 and signaling capacity is 1.

As mentioned in the caption of Figure 4b and when SHP2 is the network output node, calculated transmission error probability P_e and capacity C for the following molecules are 0 and 1, respectively: AKAp79, BAD, Bcl10, Ca, cabin1, Calcin, Calpr1, CaM, CaMK2, CaMK4, CARD11, CARD11a, Cblb, cCblp2, CD45, Cdc42, CREB, Csk, DAG, DGK, ERK, Fos, Gadd45, Gads, GAP, Grb2, GSK3, HPK1, IkB, Ikkab, Ikkg, IP3, Itk, JNK, Jun, Lckp1, Lckp2, Malt1, MEK, MEKK1, MKK4, MLK3, PAG, PDK1, PI3k, PIP3, PKB, PKCth, PLCga, PLCgb, PTEN, Rac1p1, Rac1p2, Raf, Ras, RasGRP, RlK, Rsk, sh3bp2, SHIP1, SHP1, SLP76, Sos, Vav1, Vav3.
